# HIV-related discriminatory attitudes and associated factors among pre-university students

**DOI:** 10.4102/sajhivmed.v26i1.1717

**Published:** 2025-09-22

**Authors:** Nurul F. Aziz, Fatimah Ahmad Fauzi, Rosliza Abdul Manaf

**Affiliations:** 1 Department of Community Health, Faculty of Medicine and Health Sciences, Universiti Putra Malaysia, Serdang, Malaysia

**Keywords:** HIV, discriminatory attitude, youth, risk factor, Malaysia

## Abstract

**Background:**

HIV-related stigma and discrimination are significant barriers to public health interventions, particularly among youth. In Malaysia, discriminatory attitudes towards people living with HIV (PLHIV) hinder efforts to achieve the National Strategic Plan for Ending AIDS by 2030. Stigma deters individuals from HIV testing, disclosure, treatment-seeking, and antiretroviral therapy adherence, undermining the cascade of care needed to reach the Joint United Nations Programme on HIV/AIDS (UNAIDS) 95-95-95 goals.

**Objectives:**

This study aimed to identify risk factors for HIV-related discriminatory attitudes among pre-university students.

**Method:**

A cross-sectional study was conducted at a public foundation centre in Selangor. A total of 329 pre-university students were recruited via simple random sampling. The study included active students who could read and write in Malay. Data were collected using a self-administered questionnaire. Descriptive and multivariate logistic regression analyses were conducted using SPSS version 29.0.

**Results:**

Among the 329 participants, 224 (68.1%) met the criteria for discriminatory attitudes based on global HIV stigma indicators, which assess attitudes towards interacting with PLHIV in everyday settings. Multivariate analysis identified two significant risk factors: (1) female gender (adjusted odds ratio [aOR] = 1.776, 95% confidence interval [CI] = 1.064–2.964, *P* = 0.028) and (2) inadequate HIV knowledge (aOR = 4.546, 95% CI = 2.715–7.610, *P* = 0.001).

**Conclusion:**

This study revealed a high prevalence of discriminatory attitudes among pre-university students. Female gender and inadequate HIV knowledge were significant predictors. These findings support the development of targeted interventions to reduce HIV stigma and strengthen national prevention and treatment efforts.

**What this study adds:** Female gender and inadequate HIV knowledge were key predictors of discriminatory attitudes, highlighting areas for targeted HIV stigma reduction efforts.

## Introduction

HIV-related stigma is a significant barrier to effective HIV prevention and treatment programmes worldwide. Stigma, defined as a process of devaluation, leads to discrimination and the marginalisation of people living with HIV (PLHIV). At the individual level, stigma leads to fear, social isolation, psychological distress, and avoidance of testing or treatment. Programmatically, stigma undermines public health outreach, reduces the effectiveness of prevention campaigns, creates gaps in service delivery, and contributes to disparities in HIV care access, particularly among vulnerable populations.^1^ This stigma has far-reaching effects, influencing behaviours such as delaying HIV testing, reducing treatment adherence, and decreasing overall engagement with health services.^2^ Discriminatory attitudes, driven by fear, misinformation, and cultural beliefs, further complicate efforts to control the HIV epidemic.

Globally, stigma manifests in various forms, such as anticipated stigma (the fear of experiencing discrimination), perceived stigma (awareness of negative attitudes towards PLHIV), and enacted stigma (actual experiences of discrimination).^3^ These stigma types significantly impact the lives of PLHIV, affecting their mental health, relationships, and overall quality of life. Studies indicate that stigma can exacerbate the psychological burden on PLHIV, leading to anxiety, depression, and social isolation.^4,5^

A study in Thailand found that 56.8% of youth demonstrate stigmatising attitudes toward PLHIV.^6^ In Thailand, studies have shown that stigma among youth is linked to reduced willingness to access HIV testing and education programmes, contributing to persistent transmission among key populations.^6,7^ However, most of these studies have focused on either healthcare settings or the general population, highlighting a significant research gap because of the limited focus on educational settings.

Based on the National Health and Morbidity Survey (NHMS) in 2020, 78.7% of Malaysian youth held discriminatory attitudes towards PLHIV, reflecting the pervasive nature of stigma in the region.^2^ Efforts to combat HIV stigma include the National Strategic Plan for Ending AIDS 2016–2030, which aims to reduce stigma through public health campaigns and educational programmes.^8^ As of 2023, Malaysia had an estimated 87 000 PLHIV, with a prevalence of 0.4% among adults aged 15–49. The country reported approximately 3800 new HIV infections in 2022, with sexual transmission accounting for over 90% of new cases.^9^ Malaysia’s progress towards the Joint United Nations Programme on HIV/AIDS (UNAIDS) 95-95-95 targets stands at 81% diagnosed, 68% on antiretroviral therapy, and 87% achieving viral suppression, indicating significant gaps in treatment adherence and viral load monitoring.^9^ These shortfalls may be exacerbated by HIV-related stigma and discrimination, which deter individuals from accessing services across the HIV care continuum.

Stigma remains a persistent issue, particularly among Malaysian youth. Young people in educational settings represent a critical group for HIV prevention strategies, as they are at a formative stage in developing long-term beliefs and attitudes toward HIV. This study focuses on a pre-university science foundation programme located in Serdang, within the urban Klang Valley. The centre enrols approximately 500–600 students annually from across the country, representing a diverse academic and socio-demographic profile of Malaysian youth. Given its educational mandate and national reach, this centre provides a strategic opportunity for early intervention and targeted HIV stigma reduction efforts among adolescents transitioning into higher education. The objective of this study is to identify risk factors for HIV-related discriminatory attitudes among pre-university students, with the aim of informing stigma-reduction strategies aligned with national efforts to improve HIV prevention and care outcomes.

## Research methods and design

### Study design and setting

The study was conducted at a pre-university foundation centre located in Serdang, Selangor. The centre serves students from across Malaysia and represents a cross-section of the nation’s youth population, particularly those pursuing science-based tertiary education. At the time of study, no international students or non-citizen populations were enrolled in the programme. The foundation centre is a medium-sized, urban-based academic foundation programme. Students are admitted through national-level selection processes, with representation from various states in Malaysia. The setting provides a controlled and relevant environment to examine youth attitudes on public health issues, including HIV-related stigma.

This was a single-population, quantitative cross-sectional study conducted between June and September 2024. The study population comprised registered pre-university students who were selected through simple random sampling. No comparative group was used. All participants were part of the same institutional cohort, and subgroup analyses (e.g. by gender, income, or knowledge levels) were conducted within this single sample. A sample size of 343 was determined for the HIV knowledge factor using the two independent proportions formula.^10^ A total of 329 pre-university students were recruited and willingly participated, identified through simple random sampling from a pool of registered and active students proficient in the Malay language.

### Participants and data collection

Eligible participants were aged between 17 years and 20 years and able to read and write in Malay, as the questionnaire was designed and validated in this language. Students who were unavailable during the data collection period (e.g. on leave) were excluded from the study. HIV-related discriminatory attitudes were assessed using the UNAIDS global indicator tool, which includes two standardised questions recommended by UNAIDS for measuring stigma at the population level. Respondents were asked: (1) ‘Would you buy fresh vegetables from a shopkeeper or vendor if you knew that this person had HIV?’ and (2) ‘Do you think that children living with HIV should be allowed to attend school with children who do not have HIV?’ A discriminatory attitude was recorded if a respondent answered ‘No’ to either the first or the second question. These items are globally validated and used in multiple country-level stigma surveillance efforts.^11^ Data were collected using a structured, self-administered questionnaire in Bahasa Malaysia. The questionnaire underwent face validity testing with 10 pre-university students, and content validity was assessed by two public health professionals, resulting in a content validity index (CVI) of 0.90. Reliability was confirmed through a pretest involving 30 students, with Cronbach’s alpha scores of 0.84 for the HIV knowledge section and 0.81 for the discriminatory attitudes section, indicating good internal consistency.

### Data analysis

Descriptive statistics were calculated for demographic characteristics and levels of HIV-related discriminatory attitudes. Logistic regression analysis was used to identify factors associated with discriminatory attitudes. Adjusted odds ratios (aOR) with 95% confidence intervals (CI) were calculated to assess the significance of each factor. Data analysis was performed using SPSS version 29.0 (IBM Corp., Armonk, New York, United States), with statistical significance set at *P* < 0.05.

### Ethical considerations

Ethical approval for this study was obtained from the University Putra Malaysia Ethics Review Committee for research involving human subjects under the reference number JKEUPM-2024-162. In Malaysia, the legal age of consent for participation in non-clinical research is 18 years. As participants were pre-university students aged above 18 years, written informed consent was obtained from all participants. All participants were briefed on their rights and the confidentiality of their responses before data collection.

## Results

The sociodemographic profiles of the 329 respondents are summarised in Table 1. The majority of the respondents were between 17 years and 20 years old, with a median age of 19 years (interquartile range [IQR] = 0). The sample was predominantly female. Regarding household income, 41.9% of respondents were from households earning less than Malaysian ringgit currency (RM) 5250 per month, placing them in the bottom 40% (B40) income group.

**TABLE 1:** Descriptive statistic of sociodemographic characteristic among pre-university students in Universiti Putra Malaysia (*N* = 329).

Variables	Median	IQR	*n*	%
**Age group (years)**	19.00	0.00	-	-
17–20	-	-	325	98.8
21–24	-	-	4	1.2
**Gender**						
Male	-	-	112	34.0
Female	-	-	217	66.0
**Ethnicity**						
Malaysian people	-	-	279	84.8
Chinese people	-	-	22	6.7
Indian people	-	-	19	5.8
Other†	-	-	9	2.7
**Religion**						
Islam	-	-	290	88.1
Christian	-	-	15	4.6
Hinduism	-	-	11	3.3
Buddhism	-	-	13	4.0
Other religion	-	-	0	0.0
**Residing area**						
Urban	-	-	239	72.6
Rural	-	-	90	27.4
**Household monthly income (RM)**	7000.00	6500.00	-	-
< 5250.00	-	-	138	41.9
5251.00–11 819.00	-	-	120	36.5
> 11 820.00	-	-	71	21.6

IQR, interquartile range; RM, Malaysian ringgit currency.

† , other ethnicity including people from Bugis, Kadazan and Dusun.

Among the 329 participants, 224 (68.1%) met the criteria for discriminatory attitudes based on global HIV stigma indicators, which assess attitudes toward interacting with individuals living with HIV in everyday settings.^11^ The high prevalence of discriminatory attitudes highlights the pervasive stigma that exists, even among educated youth in a university setting.

The bivariate associations between various sociodemographic factors and discriminatory attitudes are presented in Table 2. Additional personal-level factors such as HIV knowledge, HIV-related perception, media exposure, and interpersonal communication were also analysed for their association with discriminatory attitudes (Table 3). The results of the multiple logistic regression analysis identified two significant predictors of discriminatory attitudes: female gender and inadequate HIV knowledge. These factors remained significant even after adjusting for other sociodemographic variables as shown in Table 4.

**TABLE 2:** The association between sociodemographic factors and discriminatory attitudes among pre-university students in Universiti Putra Malaysia.

Socio-demographic factor	*B*	s.e.	Wald statistic	OR	95% CI	*P*
**Age group (years)**						
17–20	0.768	1.007	0.581	2.155	0.299 – 15.515	0.446
21–24	Ref	-	-	-	-	-
**Gender**						
Female	0.504	0.245	4.211	1.655	1.023 – 2.677	0.040*
Male	Ref	-	-	-	-	-
**Ethnicity**						
Malay	0.115	0.719	0.026	1.122	0.274 – 4.591	0.873
Chinese	−0.325	0.829	0.154	0.722	0.142 – 3.670	0.695
Indian	−0.154	0.852	0.033	0.857	0.161 – 4.554	0.856
Others	Ref	-	-	-	-	-
**Religion**						
Islam	0.969	0.571	2.882	2.635	0.861 – 8.064	0.090
Christian	1.166	0.806	2.089	3.208	0.660 – 15.587	0.148
Hindu	0.336	0.822	0.167	1.400	0.279 – 7.016	0.682
Buddha	−0.540	0.556	0.077	0.857	0.550 – 7.054	0.782
Others	Ref	-	-	-	-	-
**Residing area**						
Rural	0.658	0.287	5.253	1.932	1.100 – 3.393	0.022*
Urban	Ref	-	-	-	-	-
**Household monthly income (RM)**						
< 5250.00	0.566	0.319	3.151	1.761	0.943 – 3.289	0.076
5251.00–11 819.00	0.297	0.294	1.019	1.346	0.756 – 2.397	0.313
> 11 820.00	Ref	-	-	-	-	-

CI, confidence interval; OR, odds ratio; Ref, reference; RM, Malaysian ringgit currency; s.e., standard error.

* , statistical significance, *P* < 0.05.

**TABLE 3:** The association between personal factors and discriminatory attitudes among pre-university students in Universiti Putra Malaysia.

Personal factor	*B*	s.e.	Wald statistic	OR	95% CI	*P*
**Knowledge of HIV**						
Inadequate	1.485	0.260	32.734	4.417	2.655–7.347	< 0.001*
Adequate	Ref	-	-	-	-	-
**Personal perception**	0.480	0.238	4.060	1.616	1.013–2.577	0.044*
**Media exposure**	0.603	0.245	6.055	1.827	1.130–2.953	0.014*
**Interpersonal communication**	0.058	0.248	0.054	1.060	0.651–1.724	0.816

CI, confidence interval; OR, odds ratio; Ref, reference; s.e., standard error.

* , statistically significant at *P* < 0.05.

**TABLE 4:** The risk factor of discriminatory attitudes among pre-university students in Universiti Putra Malaysia.

Variable	*B*	s.e.	Wald statistic	Adjusted OR	95% CI	*P*
**Gender**						
Female	0.574	0.261	4.831	1.776	1.064–2.964	0.028*
Male	Ref	-	-	-	-	-
**Knowledge of HIV**						
Inadequate	1.514	0.263	33.156	4.546	2.715–7.610	< 0.001*
Adequate	Ref	-	-	-	-	-

CI, confidence interval; OR, odds ratio; Ref, reference; s.e., standard error.

* , statistically significant at *P* < 0.05.

Female students were more likely to exhibit discriminatory attitudes compared to their male counterparts. The odds of holding discriminatory attitudes were 1.776 times higher among female students (95% CI: 1.064–2.964, *P* = 0.028) than male students.

Inadequate knowledge of HIV was strongly associated with discriminatory attitudes. Students with inadequate HIV knowledge were 4.546 times more likely to exhibit discriminatory attitudes (95% CI: 2.715 – 7.610, *P* < 0.001) compared to those with adequate knowledge. The majority of the respondents were unable to answer all five questions correctly, indicating inadequate knowledge of HIV (*n* = 234; 71.1%).

## Discussion

The prevalence of discriminatory attitudes toward PLHIV (68.1%) in this study is higher than similar studies conducted in Thailand, where the youth stigma level was reported to be 56.8%.^6^ These studies highlight that while HIV stigma is widespread across Southeast Asia, the specific cultural, religious, and societal norms of each country play a role in shaping attitudes. Unlike Thailand, Malaysia’s diverse religious background and conservative values may contribute to the higher levels of stigma observed in this study. Additionally, fewer studies in the region have focused specifically on educational settings, where youth are in the process of forming their long-term attitudes about health and disease.^12^

The finding that female students are more likely to harbour discriminatory attitudes is consistent with previous research in Malaysia and similar settings.^13^ The implications of this finding warrant closer attention, as gender norms and societal expectations play a critical role in shaping attitudes towards health and sexuality. Cultural and societal norms often place greater emphasis on conservative views regarding sexuality and health among women, which may contribute to their stigmatising attitudes towards HIV. Women in many cultures are often less exposed to discussions about HIV and sexual health, resulting in lower HIV-related knowledge levels, further fueling stigma.^14^ In addition, stigma among women may be influenced by gender roles and expectations, which contribute to their perceptions of HIV as a morally or socially unacceptable condition.^13^

Discriminatory attitudes among youth have far-reaching implications beyond immediate social exclusion. At the programmatic level, stigma has been consistently shown to reduce willingness to undergo HIV testing due to fear of judgment or confidentiality breaches.^14^ Among those diagnosed, stigma contributes to delayed or incomplete HIV disclosure, particularly in social or family contexts, further limiting access to care and social support.^15^ In terms of treatment, internalised or enacted stigma has been associated with suboptimal adherence to antiretroviral therapy, disengagement from follow-up care, and reduced viral suppression.^5^ These outcomes reinforce the need to address stigma at its roots including among adolescents and students through structured, evidence-based educational initiatives

The strong association between inadequate HIV knowledge and discriminatory attitudes underscores the critical role that misinformation plays in perpetuating stigma. Misconceptions about how HIV is transmitted and treated can lead to irrational fears and negative judgments about PLHIV. Public health campaigns must focus on dispelling these myths and providing accurate, accessible information about HIV.^1^ Education that corrects misunderstandings around HIV transmission and addresses stigmatising beliefs can foster a more supportive attitude towards PLHIV.^16^

Programmes utilising digital tools, such as artificial intelligence (AI)-driven learning platforms, have shown promise in engaging young people and providing tailored information that can combat HIV stigma.^17^ These platforms could use interactive simulations, quizzes, and virtual peer educators to engage students in a way that traditional education methods may not. Peer-led interventions, where youth educate each other on HIV-related issues, have also been successful in promoting empathy and reducing stigma.^7^ Incorporating HIV education into broader sexual health curricula can lead to normalised discussions around HIV and reduce stigma among students.

These findings align with Malaysia’s National Strategic Plan for Ending AIDS, which emphasises the importance of reducing stigma as part of its HIV prevention and treatment efforts. By targeting youth populations, particularly those in educational settings, public health campaigns can address the root causes of stigma and create more supportive environments for PLHIV.^8^

This study has several strengths, including a robust sampling method and the use of validated questionnaires. However, there are limitations. First, discriminatory attitudes were assessed using only two questions from the UNAIDS global indicators, which may not fully capture the breadth of HIV stigma.^11^ Future studies should include a more comprehensive measure of stigma. Age group comparison in this study was limited by the small number of respondents aged 21 years – 24 years. As a result, statistical power for this comparison was low, and age was not retained in the final multivariate analysis. The inclusion of only Malay-speaking students may have excluded individuals less proficient in the language, potentially limiting generalisability and introducing selection bias. The gender distribution of respondents, which reflects the actual student enrolment during the study period, was skewed towards female students. Although adjusted analyses confirmed the independent effect of gender, this imbalance may limit the generalisability of the association between gender and discriminatory attitudes in other settings. Additionally, the study was limited to one educational institution, which may affect the generalisability of the results.

## Conclusion

This study revealed a high prevalence of discriminatory attitudes toward PLHIV among pre-university students. Female gender and inadequate HIV knowledge were identified as statistically significant predictors of stigma. These findings highlight the urgent need for comprehensive, gender-sensitive, and evidence-based interventions focused on improving HIV knowledge and challenging stigma-related misconceptions among Malaysian youth. Addressing stigma at this formative educational stage is critical to strengthening HIV prevention and treatment outcomes aligned with Malaysia’s National Strategic Plan for Ending AIDS by 2030. AI-driven educational tools and peer-led initiatives may provide effective platforms for reducing stigma, ultimately supporting Malaysia’s goal of ending AIDS by 2030.
